# Supplementary material for: Modelling the Spatial Distribution of ASF-Positive Wild Boar Carcasses in South Korea Using 2019–2020 National Surveillance Data
Source: Animals (Basel). 2021 Apr 22;11(5):1208. doi: 10.3390/ani11051208 (PMC8145688; doi:10.3390/ani11051208)
Supplement: Supplementary file 1 [file animals-11-01208-s001.zip › animals-1160614-supplementary.pdf]

Supplementary Information

Modelling the spatial distribution of ASF-infected wild boars in South Korea using 2019–2020 national surveillance data

Jun-Sik Lim[a], Timothée Vergne[b], Son-Il Pak[a], Eutteum Kim[a*]

[a]College of Veterinary Medicine and Institute of Veterinary Science, Kangwon National University, Chuncheon, Republic of Korea

[b]UMR ENVT-INRAE 1225, Ecole Nationale Vétérinaire de Toulouse, Toulouse, France

* Correspondence to eutteum@hanmail.net

1. Univariable analysis

All variables were tested independently using the Poisson and the logistic parts of the ZIP regression model. For both time periods, besides habitat suitability of wild boar, the spatial and non-spatial random terms were included in the logistic part of the regression analysis. For the second study period, the spatiotemporal and temporal autocorrelations were adjusted; this was achieved using the binary variable, indicating whether ASF-positive carcasses were reported in the first study period in the hexagon and their neighborhoods (ST_variable), and the continuous variable for the number of reports during the first study period in the hexagon (T_report) as a confounder in the logistic and Poisson parts, respectively. The results for both periods were in Table S1 and Table S2, respectively. Variables associated with at least one coefficient for which zero was not included in the 80% credible interval (CrI) of its posterior distribution were included in the multivariable analysis.

2. Correlation analysis

Collinearity between the variables was assessed using the Kendall rank correlation test [1]., The results are in Table S3 and S4. One of the variables in the pair with coefficient > 0.7 was included in the univariable analysis.

3. Spatial Zero-inflated Poisson model

With a forward selection procedure, the final multivariable models were built. Among the estimated coefficients, the estimates of spatial components including spatially structured and unstructured random effect were in Table S5.

Table S1. Results of univariable analyses for the first period (Oct. 02, 2019 ~ Jan. 18, 2020)

Variables	Categories	Number of hexagons	Only in logistic part		Only in Poisson part		Both in Poisson and logistic part			
			Odds ratio	80% CrI*	Incidence rate ratio	80% CrI*	Odds ratio	80% CrI*	Incidence rate ratio	80% CrI*
Area of water (km^2)	[0 – 0.01)	408	Ref.		Ref.		Ref.		Ref.	
	[0.01 – 0.11)	408	0.29	0.29 − 2.22	0.50	0.50 − 1.16	0.87	0.33 – 2.15	0.42	0.29 – 0.57
	[0.11 – 4.40]	421	0.21	0.21 − 1.84	0.62	0.62 − 1.38	1.47	0.54 – 3.78	1.62	1.39 – 1.89
Area of wetland	None	834	Ref.		Ref.		Ref.		Ref.	
	Presence	403	1.37	0.47 – 3.86	2.28	1.57 – 3.35	0.34	0.10 – 0.95	1.01	0.84 – 1.21
Distance to North Korea (km)	[0 – 14.62)	408	Ref.		Ref.		Ref.		Ref.	
	[14.62 – 31.53)	408	0.03	0.03 − 0.42	0.02	0.02 − 0.20	0.28	0.04 – 1.16	0.47	0.33 – 0.65
	[31.53 – 76.77]	421	0.00	0.00 − 0.11	0.00	0.00 − 0.08	0.03	0.00 – 1.61	1.83	1.57 – 2.15
Elevation (km)	[0 – 0.20)	408	Ref.		Ref.		Ref.		Ref.	
	[0.20 – 0.38)	408	0.21	0.21 − 3.72	0.30	0.30 − 0.94	0.76	0.22 – 2.48	0.58	0.41 – 0.8
	[0.38 – 1.16]	421	0.14	0.14 − 5.91	1.35	1.35 − 10.71	0.33	0.06 – 1.52	1.02	0.86 – 1.21
Slope (meters)	[0.19 – 13.89)	408	Ref.		Ref.		Ref.		Ref.	
	[13.89 – 21.32)	408	0.63	0.63 − 7.4	0.36	0.36 − 1.01	1.03	0.31 – 3.08	0.46	0.31 – 0.63
	[21.32 – 31.65]	421	0.08	0.08 − 3.55	0.03	0.03 − 0.34	1.12	0.27 – 4.33	0.86	0.72 – 1.03
Heat load index	[0.62 – 0.81)	408	Ref.		Ref.		Ref.		Ref.	
	[0.81 – 0.84)	408	0.13	0.13 − 1.43	1.07	1.07 − 2.54	0.21	0.06 – 0.61	1.04	0.74 – 1.44

Table S1. Results of univariable analyses for the first period (Oct. 02, 2019 ~ Jan. 18, 2020)

Variables	Categories	Number of hexagons	Only in logistic part		Only in Poisson part		Both in Poisson and logistic part			
			Odds ratio	80% CrI*	Incidence rate ratio	80% CrI*	Odds ratio	80% CrI*	Incidence rate ratio	80% CrI*
	[0.84 – 0.99]	421	0.28	0.28 − 3.04	0.22	0.22 − 0.65	0.73	0.25 – 1.9	3.05	2.38 – 3.95
Area of rice paddy (km^2)	[0 – 0.01)	408	Ref.		Ref.		Ref.		Ref.	
	[0.01 – 0.05)	408	0.19	0.19 − 3.10	0.06	0.06 − 0.47	1.91	0.65 – 5.4	0.42	0.29 – 0.59
	[0.05 – 7.18]	421	1.07	1.07 − 15.07	0.19	0.19 − 1.86	2.6	0.79 – 8.13	1.24	1.04 – 1.5
Human population (person per km^2)	[15.68 – 24.77)	408	Ref.		Ref.		Ref.		Ref.	
	[24.77 – 72.36)	408	0.11	0.11 − 3.13	1.35	1.35 − 10.60	0.03	0.01 – 0.14	0.41	0.28 – 0.58
	[72.36 – 9448.67]	421	0.00	0.00 − 0.09	2.55	2.55 − 21.96	0.00	0 – 0.02	0.71	0.55 – 0.9
Land surface temperature at day (°C)	[9.01 – 13.10)	408	Ref.		Ref.		Ref.		Ref.	
	[13.10 – 15.17)	408	0.01	0.01 − 0.33	0.02	0.02 − 0.21	3.81	1.32 – 11.51	0.58	0.41 – 0.78
	[15.17 – 20.27]	421	0.04	0.04 − 2.05	0.15	0.15 − 9.89	0.55	0.11 – 2.36	1.02	0.84 – 1.23
Enhanced Vegetation Index	[0.02 – 0.15)	408	Ref.		Ref.		Ref.		Ref.	
	[0.15 – 0.18)	408	0.42	0.42 − 2.82	0.7	0.7 − 1.6	0.71	0.26 – 1.79	0.44	0.29 – 0.64
	[0.18 – 0.29]	421	0.24	0.24 − 3.16	0.22	0.22 − 0.72	0.89	0.23 – 3.18	0.81	0.67 – 0.97
Normalized Difference Water Index	[-1.00 – -0.29)	408	Ref.		Ref.		Ref.		Ref.	
	[-0.29 – -0.04)	408	0.01	0.01 − 0.24	0.90	0.90 − 30.39	0.42	0.15 – 1.09	0.34	0.23 – 0.49

Table S1. Results of univariable analyses for the first period (Oct. 02, 2019 ~ Jan. 18, 2020)

Variables	Categories	Number of hexagons	Only in logistic part		Only in Poisson part		Both in Poisson and logistic part			
			Odds ratio	80% CrI*	Incidence rate ratio	80% CrI*	Odds ratio	80% CrI*	Incidence rate ratio	80% CrI*
	[-0.04 – 0.80)	421	0.09	0.09 − 2.23	0.35	0.35 − 1.64	0.19	0.03 – 0.85	2.05	1.71 – 2.46
Land surface temperature at night (°C)	[1.89 – 5.52)	408	Ref.		Ref.		Ref.		Ref.	
	[5.52 – 6.42)	408	0.26	0.26 − 1.79	0.74	0.74 − 1.56	0.86	0.32 – 2.17	0.53	0.38 – 0.73
	[6.42 – 12.41]	421	0.05	0.05 − 0.98	0.46	0.46 − 1.75	1.04	0.32 – 3.16	1.22	1.05 – 1.43
Rainfall (mm)	[42.44 – 51.43)	408	Ref.		Ref.		Ref.		Ref.	
	[51.43 – 57.27)	408	0.05	0.05 − 0.78	1.23	1.23 − 2.60	0.18	0.03 – 1.00	0.47	0.33 – 0.66
	[57.27 – 150.81]	421	0.07	0.07 − 5.76	3.73	3.73 − 42.82	0.2	0.02 – 1.58	0.81	0.62 – 1.03

*CrI: Credible interval

Table S2. Results of univariable analyses for the second period (Jan. 19, 2020 ~ Apr. 28, 2020)

Variables	Category	Number of hexagons	Only in logistic part		Only in Poisson part		Both in Poisson and logistic part			
			Odds ratio	80% CrI*	Incidence rate ratio	80% CrI*	Odds ratio	80% CrI*	Incidence rate ratio	80% CrI*
Area of water (km^2)	[0 – 0.01)	408	Ref.		Ref.		Ref.		Ref.	
	[0.01 – 0.11)	408	0.97	0.37 – 2.41	1.61	1.39 – 1.88	0.86	0.26 − 2.35	1.23	1.03 − 1.46
	[0.11 – 4.40]	421	1.41	0.52 – 3.61	0.75	0.64 – 0.89	2.54	0.72 − 8.26	0.72	0.58 − 0.88
Area of wetland	None	834	Ref.		Ref.		Ref.		Ref.	
	Presence	403	0.35	0.11 – 0.96	1.00	0.85 – 1.20	0.33	0.11 – 0.95	1.01	0.84 – 1.21
Distance to North Korea (km)	[0 – 14.62)	408	Ref.		Ref.		Ref.		Ref.	
	[14.62 – 31.53)	408	0.45	0.11 – 1.58	1.84	1.57 – 2.16	0.34	0.08 − 1.19	1.84	1.56 − 2.17
	[31.53 – 76.77]	421	0.01	0 – 0.06	0.01	0 – 0.05	0.02	0.00 − 0.36	0.15	0.00 − 29.14
Elevation (km)	[0 – 0.20)	408	Ref.		Ref.		Ref.		Ref.	
	[0.20 – 0.38)	408	0.77	0.23 – 2.49	1.01	0.85 – 1.21	0.81	0.25 − 2.43	1.01	0.84 − 1.19
	[0.38 – 1.16]	421	0.33	0.06 – 1.46	0.8	0.64 – 1.01	0.36	0.07 − 1.53	0.79	0.64 − 0.99
Slope (meters)	[0.19 – 13.89)	408	Ref.		Ref.		Ref.		Ref.	
	[13.89 – 21.32)	408	0.95	0.29 – 2.88	0.86	0.72 – 1.03	0.94	0.28 − 3.09	0.86	0.72 − 1.03
	[21.32 – 31.65]	421	1.08	0.27 – 4.18	1.15	0.93 – 1.44	1.11	0.25 − 4.62	1.15	0.94 − 1.42
Heat load index	[0.62 – 0.81)	408	Ref.		Ref.		Ref.		Ref.	
	[0.81 – 0.84)	408	0.32	0.1 – 0.85	2.91	2.29 – 3.74	0.18	0.04 − 0.55	3.10	2.4 – 4.00

Table S2. Results of univariable analyses for the second period (Jan. 19, 2020 ~ Apr. 28, 2020)

Variables	Category	Number of hexagons	Only in logistic part		Only in Poisson part		Both in Poisson and logistic part			
			Odds ratio	80% CrI*	Incidence rate ratio	80% CrI*	Odds ratio	80% CrI*	Incidence rate ratio	80% CrI*
	[0.84 – 0.99]	421	1.00	0.39 – 2.53	2.67	2.12 – 3.37	0.68	0.23 − 1.85	2.81	2.2 − 3.55
Area of rice paddy (km^2)	[0 – 0.01)	408	Ref.		Ref.		Ref.		Ref.	
	[0.01 – 0.05)	408	2.04	0.73 – 5.57	1.26	1.05 – 1.52	2.06	0.75 − 5.89	1.08	0.91 − 1.31
	[0.05 – 7.18]	421	2.6	0.84 – 7.86	0.81	0.64 – 1.04	2.74	0.86 − 8.87	0.69	0.55 − 0.88
Human population (person per km^2)	[15.68 – 24.77)	408	Ref.		Ref.		Ref.		Ref.	
	[24.77 – 72.36)	408	0.03	0.01 – 0.13	0.70	0.54 – 0.89	0.03	0.01 − 0.12	0.72	0.56 − 0.92
	[72.36 – 9448.67]	421	0.00	0.00 – 0.02	0.61	0.45 – 0.81	0.00	0.00 − 0.02	0.64	0.47 − 0.86
Day Land surface temperature (°C)	[-1.93 – 3.56)	408	Ref.		Ref.		Ref.		Ref.	
	[3.56 – 5.39)	408	3.73	1.26 – 11.76	1.03	0.84 – 1.26	3.66	1.2 − 11.79	1.02	0.84 − 1.24
	[5.39 – 9.32]	421	0.53	0.1 – 2.34	1.19	0.95 – 1.5	0.49	0.11 − 2.32	1.18	0.96 − 1.5
Enhanced Vegetation Index	[0.02 – 0.15)	408	Ref.		Ref.		Ref.		Ref.	
	[0.15 – 0.18)	408	0.64	0.23 – 1.65	0.81	0.67 – 0.97	0.67	0.23 − 1.79	0.81	0.67 − 0.98
	[0.18 – 0.29]	421	0.95	0.25 – 3.4	1.21	0.94 – 1.55	0.82	0.2 − 3.15	1.19	0.93 − 1.53
Normalized Difference Water Index	[-0.90 – -0.41)	408	Ref.		Ref.		Ref.		Ref.	
	[-0.41 – -0.25)	408	0.47	0.16 – 1.21	2.05	1.72 – 2.45	0.25	0.08 − 0.75	0.85	0.68 − 1.04
	[-0.25 – 1]	421	0.16	0.03 – 0.75	0.85	0.57 – 1.25	0.07	0.01 − 0.38	0.58	0.4 − 0.8

Table S2. Results of univariable analyses for the second period (Jan. 19, 2020 ~ Apr. 28, 2020)

Variables	Category	Number of hexagons	Only in logistic part		Only in Poisson part		Both in Poisson and logistic part			
			**Odds ratio**	**80% CrI***	**Incidence rate ratio**	**80% CrI***	**Odds ratio**	**80% CrI***	**Incidence rate ratio**	**80% CrI***
Night Land surface temperature (°C)	[-5.23 – -1.73)	408	Ref.		Ref.		Ref.		Ref.	
	[-1.73 – -0.72)	408	0.88	0.34 – 2.23	1.23	1.06 – 1.42	0.90	0.38 − 2.14	1.21	1.05 − 1.41
	[-0.72 – 4.78]	421	1.07	0.33 – 3.23	1.19	1.00 – 1.4	1.09	0.37 − 3.11	1.17	1.01 − 1.38
Rainfall (mm)	[26.65 – 33.52)	408	Ref.		Ref.		Ref.		Ref.	
	[33.52 – 35.88)	408	0.19	0.03 – 0.95	0.81	0.62 – 1.02	0.20	0.03 − 0.9	0.81	0.64 − 1.01
	[35.88 – 48.96]	421	0.18	0.02 – 1.37	0.63	0.45 – 0.86	0.19	0.02 − 1.38	0.64	0.47 − 0.86

*CrI: Credible interval

Table S3. Kendall Correlation coefficients between the variables significant at 20% in univariable analysis for the first period (Oct. 02, 2019 ~ Jan. 18, 2020)

	Area_water[1]	DNorthKor[2]	Elevation	Slope	HLI[3]	Area_rice[4]	Hu_pop[5]	LSTD[6]	EVI[7]	NDWI[8]	LSTN[9]	Precp[10]
Area_water[1]	1.00	0.04	-0.25	-0.20	-0.09	0.24	0.14	0.17	-0.11	-0.05	0.07	-0.14
DNorthKor[2]		1.00	0.08	0.18	0.00	-0.09	0.08	-0.21	0.23	0.41	0.19	0.02
Elevation			1.00	0.67	0.16	-0.60	-0.52	-0.73	0.17	0.18	-0.27	0.31
Slope				1.00	0.16	-0.64	-0.39	-0.70	0.23	0.19	-0.01	0.29
HLI[3]					1.00	-0.10	-0.05	-0.12	0.19	-0.03	-0.11	-0.03
Area_rice[4]						1.00	0.30	0.56	-0.17	-0.12	0.02	-0.30
Hu_pop[5]							1.00	0.52	-0.10	-0.15	0.33	-0.36
LSTD[6]								1.00	-0.17	-0.29	0.17	-0.40
EVI[7]									1.00	0.21	0.15	0.06
NDWI[8]										1.00	0.17	0.24
LSTN[9]											1.00	-0.13
Rainfall												1.00

[1]Area of water: Area of water within a hexagon; [2.] DNorthKor: Distance to North Korea from the centroid of each hexagon; [3] HLI: heat load index; [4] Area_rice: Area of rice paddy within a hexagon; [5.] Hu_pop: Human population density; [6.] LSTD: Land surface temperature at day; [7]. EVI: Enhanced Vegetation Index; [8]. NDWI: Normalized Difference Water Index; [9.] LSTN: Land Surface Temperature at Night;

Table S4. Kendall Correlation coefficients between the variables significant at 20% in univariable analysis for the second period (Jan. 19, 2020 ~ Apr. 28, 2020)

	Area_water[1]	Wetland[2]	DNorthKor[3]	Elevation	HLI[4]	Area_rice[5]	Hu_pop[6]	LSTD[7]	EVI[8]	NDWI[9]	LSTN[10]	Precp[11]
Area_water[1]	1.00	0.23	-0.06	-0.46	-0.18	0.40	0.15	0.32	-0.16	-0.11	-0.07	-0.02
DNorthKor[2]		1.00	0.04	-0.25	-0.09	0.24	0.14	0.17	-0.09	-0.04	-0.07	-0.14
Elevation			1.00	0.08	0.00	-0.09	0.08	-0.18	0.32	0.42	0.09	-0.13
Slope				1.00	0.16	-0.60	-0.52	-0.75	0.22	0.20	0.01	0.01
HLI[3]					1.00	-0.10	-0.05	-0.13	0.12	-0.04	-0.12	-0.04
Area_rice[4]						1.00	0.30	0.54	-0.11	-0.13	-0.06	-0.03
Hu_pop[5]							1.00	0.54	-0.20	-0.18	0.11	-0.13
LSTD[6]								1.00	-0.20	-0.26	0.09	0.09
EVI[7]									1.00	0.42	0.10	-0.04
NDWI[8]										1.00	0.08	0.11
LSTN[9]											1.00	0.02
Rainfall												1.00

[1]Area of water: Area of water within a hexagon; [2.] DNorthKor: Distance to North Korea from the centroid of each hexagon; [3] HLI: heat load index; [4] Area_rice: Area of rice paddy within a hexagon; [5.] Hu_pop: Human population density; [6.] LSTD: Land surface temperature at day; [7]. EVI: Enhanced Vegetation Index; [8]. NDWI: Normalized Difference Water Index; [9.] LSTN: Land Surface Temperature at Night;

Table S5. The estimated variances of spatial components in spatial zero-inflated Poisson model for the first (Oct. 02, 2019 ~ Jan. 18, 2020) and second periods (Jan. 19, 2020 ~ Apr. 28, 2020)

**Values**	Period 1 **(Oct. 02, 2019 ~ Jan. 18, 2020)**	Period 2 **(Jan. 19, 2020 ~ Apr. 28, 2020)**
	Variance (95% CrI*)	Variance (95% CrI*)
Spatial random term	12.99 (2.72 – 51.14)	34.76 (14.92 – 84.02)
Non-spatial random term	0.19 (0.03 – 2.93)	0.09 (0.02 – 0.82)

*CrI: Credible interval

4. References

1. Abdi, H.J.E.o.M. and T.O. Statistics. Sage, CA, *The Kendall rank correlation coefficient.* 2007: p. 508-510.
